# Partial Phallectomy and Penile Retroversion as a Surgical Approach for Severe Preputial Laceration in a Donkey (*Equus asinus*)

**DOI:** 10.1155/crve/9036471

**Published:** 2025-07-15

**Authors:** Letícia Paranhos Rios Andrade, Helena Tavares Dutra, Jéssyca Ataíde Ferreira, Jéssica Sola Quirino da Silva, Mariana Quinan Bittar, Paulo José Bastos Queiroz

**Affiliations:** ^1^School of Veterinary Medicine and Animal Science, Federal University of Goiás, Goiânia, Goiás, Brazil; ^2^Equus Center Veterinary Hospital, Goiânia, Goiás, Brazil

## Abstract

Penile trauma in equines can cause injuries of varying severity, potentially leading to temporary or permanent dysfunction. In severe cases, various surgical techniques can be employed to restore the animal's quality of life, such as partial phallectomy combined with penile retroversion. However, descriptions of the application of this technique in donkeys are lacking. This report describes the surgical treatment of a donkey with extensive preputial and penile laceration using the technique of partial phallectomy combined with penile retroversion. An 18-month-old intact male donkey presented with extensive preputial and penile lacerations, resulting in the complete rupture of the preputial lamina, permanent penile protrusion, tissue necrosis, and urethral rupture with urine leakage. The donkey underwent a partial phallectomy and penile retroversion after a 2-week preoperative period, which included debridement, dressings, and antibiotic therapy. The surgical procedure involved the creation of a perineal urethrostomy and partial penile amputation. Postoperative care included antibiotic and anti-inflammatory therapy, as well as local wound management. Postoperative complications included postmicturition hemorrhage and partial suture dehiscence. Despite these issues, a complete recovery was achieved, and the donkey was discharged after 56 days postsurgery. Four years later, the donkey was urinating normally without complications. Penile retroversion combined with partial phallectomy proves to be an effective surgical approach for treating extensive penile and preputial lacerations in donkeys, providing lasting results and long-term complication-free outcomes.

## 1. Introduction

Penile trauma in equines can occur in various situations, such as attempts to jump fences, kicks from other horses or inadequately restrained mares during mating, falls onto sharp or penetrating objects, and lacerations caused by the mare's tail hairs [1]. These traumas can result in contusions or lacerations of varying severity, potentially leading to temporary or permanent penile dysfunction. In cases of severe lacerations with extensive tissue damage to the penis and prepuce, several surgical techniques may be employed to restore the animal's quality of life [2].

Partial phallectomy combined with penile retroversion is a surgical technique indicated for treating extensive injuries to the inner preputial lamina and the penis [3, 4]. This procedure is often associated with en bloc resection in cases of invasive neoplasms involving the penis, prepuce, and inguinal lymph nodes [4, 5]. However, it is an invasive approach that causes significant alterations to the external genitalia and is therefore reserved as a last resort for severe cases [1, 5].

While there are some reports of partial phallectomy combined with penile retroversion in equines [3, 5, 6], no descriptions of this technique applied to donkeys have been found. Therefore, this case report describes the surgical treatment of a donkey with extensive preputial and penile laceration using the technique of partial phallectomy combined with penile retroversion.

## 2. Case Report

A male Pega donkey, 18 months old, weighing 240 kg, with a good body condition score (7/9), was presented at the Equus Center Veterinary Hospital. According to the owner, 1 week prior to the consultation, the donkey had become entangled in a wooden fence while attempting to jump over it, resulting in extensive abdominal, preputial, and penile lacerations.

**Figure 1:**
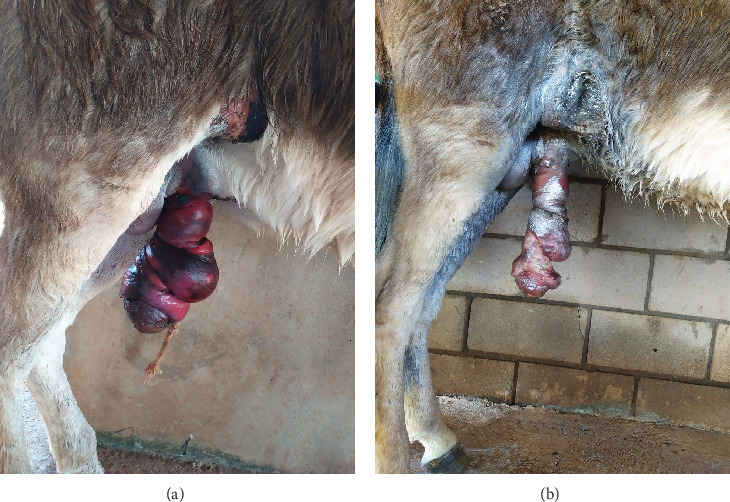
An 18-month-old intact male donkey with extensive preputial, penile, and ventrocaudal abdominal lacerations. (a) Aspect of the lesion at the initial presentation, showing edema and cyanotic discoloration of the free part of the penis and the prepuce. (b) Lesion aspect 1 week after the initial presentation, showing necrosis of the skin on the free part of the penis and the prepuce.

On clinical examination, all vital parameters were within normal ranges. The visual inspection revealed a severe ventrocaudal abdominal laceration involving the prepuce and penis, with the presence of edema and a cyanotic coloration on the free part of the penis (Figure 1a). A complete blood count revealed normocytic, normochromic anemia and leukopenia due to neutropenia. Biochemical analysis showed hypoproteinemia, hypoalbuminemia, and hypoglobulinemia.

Initially, hydrotherapy and debridement of necrotic tissue were performed, followed by daily wound dressings with 0.1% povidone-iodine, a penicillin-based ointment, and an insect-repellent spray. Antibiotic therapy with potassium penicillin (30,000 IU/kg, IV, QID) and gentamicin (6.6 mg/kg, IV, SID) was administered for 7 days, along with anti-inflammatory treatment using flunixin meglumine (1.1 mg/kg, IV, SID) for 5 days. However, after 1 week, necrosis of the penile skin and both the internal and external preputial laminae were observed (Figure 1b).

**Figure 2:**
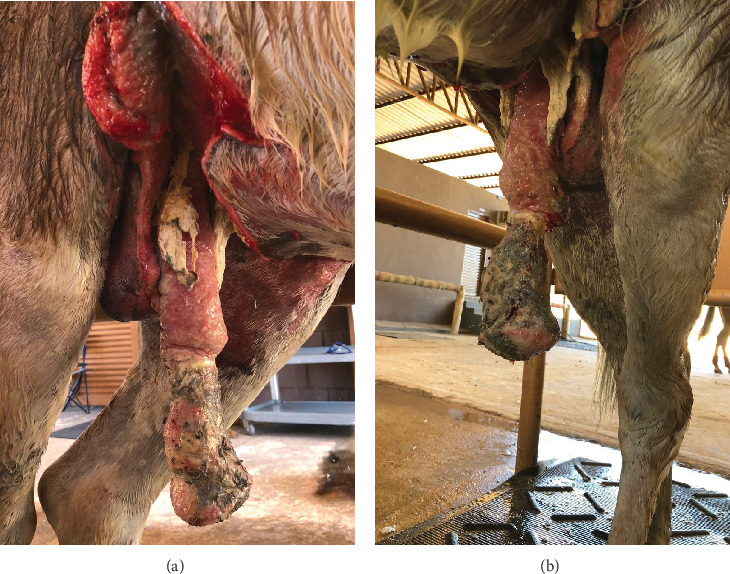
An 18-month-old intact male donkey with extensive preputial, penile, and ventrocaudal abdominal lacerations 2 weeks after the initial presentation. Necrotic tissue, purulent discharge, and granulation tissue are evident on the penis. (a) Right lateral view. (b) Left ventrolateral view.

Two weeks after the beginning of treatment, a complete rupture of the inner and outer preputial laminae was observed, leading to permanent penile protrusion. Cranial to the preputial ring, a laceration on the free portion of the penis extended into the urethral lumen, resulting in urine leakage during micturition (Supporting Information 1). Additionally, necrotic tissue, purulent discharge with a foul odor, and areas of granulation tissue were noted (Figure 2a,b).

The owner was informed of the severity of the injuries, which required surgical intervention to remove the necrotic portion of the penis in order to restore the animal's well-being. It was explained that, due to the laceration of the preputial laminae, permanent exposure of the free portion of the penis, extensive infection and necrosis of the penile skin, and rupture of the penile urethra, conservative treatment with only wound management and systemic antibiotic therapy would be ineffective and would unnecessarily prolong the animal's suffering. In light of this, the owner consented to the surgical procedure and signed the informed consent form acknowledging the associated risks.

The animal was fasted for 12 h, and potassium penicillin (30,000 IU/kg, IV), gentamicin (6.6 mg/kg, IV), and flunixin meglumine (1.1 mg/kg, IV) were administered 30 min before surgery. Preanesthetic medication included detomidine hydrochloride (0.02 mg/kg, IV); anesthesia was induced with ketamine hydrochloride (2.2 mg/kg, IV) and midazolam maleate (0.05 mg/kg, IV) and maintained with isoflurane inhalation anesthesia. Additionally, a nerve block with lidocaine hydrochloride (3.3 mg/kg) was performed on the superficial and deep branches of the left and right pudendal nerves. The patient was placed in dorsal recumbency, and the abdominal and perineal regions were prepared for surgery. A urinary catheter was inserted into the bladder.

The technique used was similar to that described by Perkins et al. [3]. A 10-cm vertical incision was made along the perineal raphe, 20 cm below the anus and ventral to the ischium. The incision was deepened through the subcutaneous fascia to visualize the retractor penis muscles, which were split and reflected laterally to expose the penis. Blunt dissection was performed to free the penile body from adjacent tissues, continuing distally to the ruptured preputial cavity. Once the penis was fully freed, it was pulled caudally through the perineal incision, and a tourniquet was placed dorsally to the proposed urethrostomy site.

**Figure 3:**
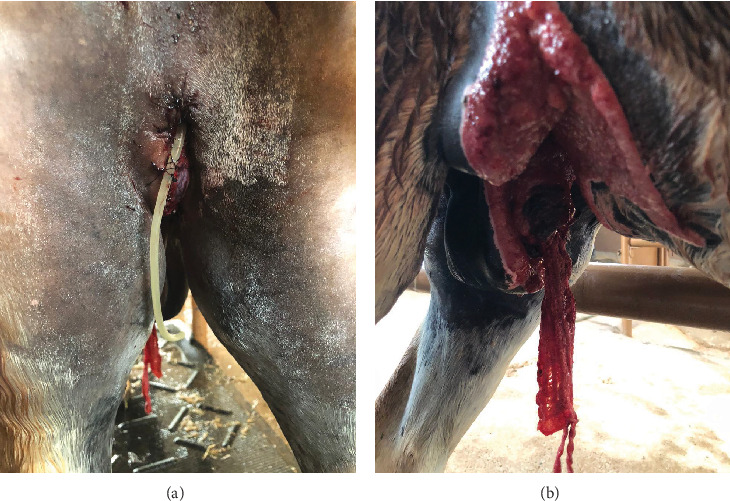
Immediate postoperative appearance following partial phallectomy and penile retroversion in a Pega donkey. (a) Urethral catheter secured at the perineal urethrostomy site to prevent potential urination difficulties. (b) Gauze pad placed in the orifice left by the penectomy to aid in hemostasis.

Partial phallectomy was performed using the Williams technique as described by Schumacher [1]. At the level of the perineal incision, a triangular urethrostomy was created on the ventral aspect of the penis, with a 3 cm base oriented distally and a height of 7 cm. The urethra was incised longitudinally and sutured laterally to the tunica albuginea with continuous simple sutures using 3-0 polydioxanone. On the dorsal aspect of the penis, the dorsal plexus arteries and veins were ligated at the proposed amputation site, and the penile body was obliquely transected at the base of the urethrostomy, extending distodorsally. The cavernous body was closed with interrupted simple sutures using 2-0 polyglactin 910, incorporating the urethral mucosa, the urethral groove tunica albuginea, and the tunica albuginea surrounding the penile cavernous body. After ensuring hemostasis, the three sides of the urethrostomy triangle were sutured to the perineal skin using simple interrupted sutures with 2-0 polyamide. A urethral catheter was maintained for 24 h after surgery to prevent potential urination difficulties (Figure 3). The abdominal wound cranial to the scrotum was left unsutured to heal by second intention. A gauze pad was inserted into the orifice left after the penectomy and maintained for 24 h to assist with hemostasis (Figure 3). The owner opted not to perform castration, intending to collect semen directly from the epididymal tail after a future castration. Anesthetic recovery was uneventful.

Postoperative care included potassium penicillin (30,000 IU/kg, IV, QID) and gentamicin (6.6 mg/kg, IV, SID) for 7 days. Anti-inflammatory and analgesic therapy included dipyrone sodium (25 mg/kg, IV, TID) for 4 days and flunixin meglumine (1.1 mg/kg, IV, SID) for 4 days. Local wound care was continued as in the preoperative period. For the perineal urethrostomy, penicillin-based ointment and ice compresses were applied for 5 days.

**Figure 4:**
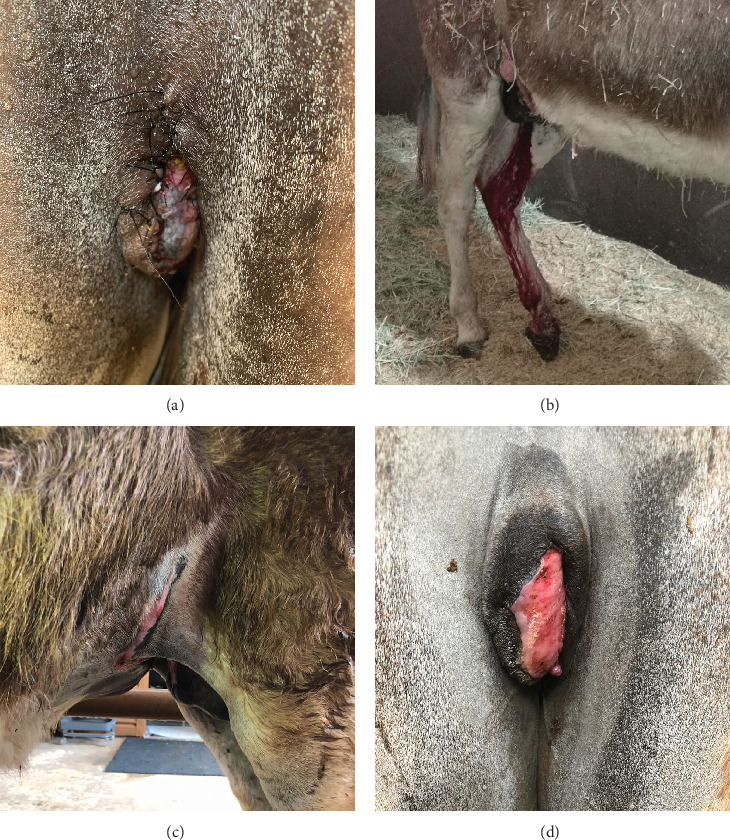
Postoperative outcomes of partial phallectomy and penile retroversion surgery in a Pega donkey. (a) Perineal urethrostomy 2 days after surgery. (b) Hemorrhage at the urethrostomy site following urination on the second postoperative day. (c) Healed abdominal wound at the time of hospital discharge. (d) Healed perineal urethrostomy at the time of hospital discharge.

Two days after surgery, the perineal urethrostomy had a good appearance (Figure 4a), and the patient was able to urinate normally, adopting a urination posture characteristic of female donkeys, without urine leakage down the hind limbs (Supporting Information 2). Postoperative complications included urethrostomy bleeding at the end of urination for 21 days (Figure 4b), edema, and partial suture dehiscence, which healed by second intention. Sutures were removed after 14 days. The left side of the urethral mucosa in the urethrostomy remained more exposed and was later covered by granulation tissue, followed by penile epithelial tissue (Figure 4c). This alteration did not impair urination. The patient was discharged 56 days postoperatively with near-complete healing of the ventral wound (Figure 4d).

Thirty-eight months after surgery, the owner was contacted via a messaging app and reported that the donkey was in good health, with no complications such as urethral meatus stenosis or urine scalding.

## 3. Discussion

This report describes the successful long-term treatment of extensive abdominal, preputial, and penile lacerations in a donkey using partial phallectomy combined with penile retroversion due to the impossibility of anatomical reconstruction with less invasive surgical techniques. No reports of this surgical technique applied to donkeys were found, likely due to their smaller population compared to horses and their lower commercial value. The population of donkeys and mules has been growing, especially in developing countries, where they are commonly used for labor [7]. This could be a contributing factor to the lower number of surgical cases in these animals. The anatomy of the penis and prepuce in donkeys is similar to that of male horses; however, donkeys have a longer penis, a less evident preputial ring, and two teats on the prepuce [8]. Thus, surgical techniques used for the reproductive system of male horses can also be applied to donkeys [9].

Partial phallectomy combined with penile retroversion was chosen due to the destruction of the inner and outer preputial laminae and the presence of penile necrosis, which precluded the use of less invasive techniques. Mild penile and preputial lacerations may be managed through primary closure when the wounds are recent, minimally contaminated, well-vascularized, exhibit moderate tissue loss, and allow for minimal tension at the wound margins [10]. However, these criteria were not met in the present case. The animal was presented for treatment 1 week after the trauma, and the lesion exhibited extensive contamination, necrosis, and significant tissue loss. In such scenarios, second-intention healing is a conservative treatment option [10] and was employed for the management of the abdominal wound after surgery, which could not be sutured. However, the complete laceration of both the internal and external preputial laminae, which prevented retraction of the penis into the preputial cavity—resulting in a case of paraphimosis—alongside the severe necrosis of the free portion of the penis, necessitated a more invasive surgical approach.

Although trauma-induced paraphimosis may be managed conservatively using a variety of techniques [1, 11, 12], these methods were not applicable in this case due to the complete rupture of both preputial laminae. Likewise, less invasive surgical procedures are generally indicated for more superficial injuries involving limited portions of the penis and prepuce. For instance, posthioplasty (reefing) is suitable for lesions confined to the skin and superficial tissues of the free portion of the penis [1, 5], and various techniques of partial phallectomy have been described for injuries affecting the glans or penile body [1]. In the present case, however, the severity and extent of the trauma—affecting both the internal and external preputial laminae as well as the penile body—precluded the use of conservative or less invasive surgical techniques. As a result, a partial phallectomy combined with penile retroversion was required, following the approach described by Perkins et al. [3].

Several publications have demonstrated the use of en bloc resection and partial phallectomy, with or without penile retroversion, for the treatment of invasive neoplasms affecting the penis, prepuce, and inguinal lymph nodes in horses [3–6, 13]. However, reports on the surgical treatment of severe lacerations of the external genitalia in male equids are scarce [3].

The preoperative bloodwork revealed normocytic, normochromic, nonregenerative anemia, which may be associated with local hemorrhage due to trauma and chronic inflammation. In horses, nonregenerative anemia is often attributed to reduced erythropoiesis caused by decreased iron availability, a result of inflammatory cytokines [14]. Leukopenia due to neutropenia was also observed, which commonly occurs due to the rapid utilization of neutrophils in severely inflamed tissues [14].

Biochemical analyses showed hypoproteinemia, hypoalbuminemia, hypoglobulinemia, and reduced serum creatinine levels. Hypoproteinemia, caused by hypoalbuminemia and hypoglobulinemia, can occur in patients with hemorrhagic trauma and exudative skin diseases [15]. Although significant hemorrhage at the time of injury was not reported, some blood loss likely occurred, considering the high vascularization of the preputial and penile region. Additionally, there was considerable exudation from the injured area, particularly during the first days of hospitalization, explaining hypoproteinemia. The reduced serum creatinine concentration may be observed in cases of muscle mass loss due to chronic cachexia [16]. In this case, the patient exhibited hyporexia, possibly associated with stress from the change in environment and diet, as the animal was pasture-raised on the property. Pain from the severe injury likely also contributed to the reduced food intake.

The postoperative therapeutic protocol included antibiotic therapy with a combination of potassium penicillin and gentamicin, as well as anti-inflammatory and analgesic therapy with flunixin meglumine and dipyrone. Penicillins are bactericidal antibiotics effective against gram-positive bacteria but with low effectiveness against gram-negative bacteria [17]. For this reason, they are often combined with aminoglycosides like gentamicin, which are bactericidal and effective against gram-negative bacteria [18]. Flunixin meglumine is a nonsteroidal anti-inflammatory drug that inhibits cyclooxygenase enzymes, blocking eicosanoid synthesis and aiding in pain control caused by inflammation [19]. Due to the significant surgical trauma, dipyrone was added to flunixin meglumine to enhance analgesia, as it has potent analgesic and antipyretic effects [20].

Postoperative complications included edema, partial suture dehiscence, and postmicturition hemorrhage for 21 days. These complications have also been observed by other authors with varying degrees of severity [5, 13]. Postmicturition hemorrhage was the most severe complication and caused concern due to its long duration, leading to anemia, tachycardia, and tachypnea. This condition is common after phallectomy in horses [5, 13, 21, 22]. The hemorrhage originates from the corpus spongiosum penis, which maintains high internal pressure following the rapid reduction of urethral intraluminal pressure after urination [1]. The maintenance of the donkey intact likely contributed to the occurrence of postmicturition hemorrhage, as castration is recommended at least 3 weeks prior to a partial phallectomy to prevent erection, which can lead to hemorrhage and dehiscence [1]. In this case, castration was not performed before the surgery due to the severity of the condition. Additionally, the donkey was not castrated during the surgery because the owner wished to collect semen directly from the epididymal tail following a future castration. Despite lasting 3 weeks, the hemorrhage resolved spontaneously, and no further intervention was necessary.

The 56-day hospitalization period exceeded that reported in other studies [3–5]. This extended period was due to the inability to perform wound care at the owner's property. Consequently, the owner opted to keep the animal hospitalized until the complete second-intention healing of the wound was achieved.

Long-term follow-up revealed that the animal remains in good health, and the owner is satisfied with the outcome. Straticó et al. [4] reported a case of a horse that underwent en bloc resection combined with perineal urethrostomy and showed no recurrence of neoplasia or additional surgical complications after 3 years.

Penile retroversion combined with a partial phallectomy is an effective surgical approach for treating extensive lacerations of the penis and prepuce in donkeys. This technique promotes animal welfare recovery and provides long-lasting results without long-term complications, making it a viable and safe option for similar cases.

## Data Availability

Data sharing is not applicable to this article as no new data were created or analyzed in this study.

## Supporting Information

Supporting Information 1 An 18-month-old intact male donkey presented with extensive penile laceration and necrosis. The video shows necrosis of the free portion of the penis and rupture of the urethra, resulting in urine leakage.

Supporting Information 2 An 18-month-old male donkey following partial phallectomy and penile retroversion. The video demonstrates the animal adopting a female-like urination posture, with effective urinary outflow and no evidence of urine leakage along the hind limbs.
